# Wnt/β-catenin pathway regulates *ABCB1* transcription in chronic myeloid leukemia

**DOI:** 10.1186/1471-2407-12-303

**Published:** 2012-07-23

**Authors:** Stephany Corrêa, Renata Binato, Bárbara Du Rocher, Morgana TL Castelo-Branco, Luciana Pizzatti, Eliana Abdelhay

**Affiliations:** 1 Divisão de Laboratórios CEMO, INCA, Rio de Janeiro, Brazil; 2 Instituto de Biofísica Carlos Chagas Filho, Universidade Federal do Rio de Janeiro, Rio de Janeiro, Brazil; 3 Instituto de Ciências Biomédicas, Universidade Federal do Rio de Janeiro, Rio de Janeiro, Brazil

## Abstract

**Background:**

The advanced phases of chronic myeloid leukemia (CML) are known to be more resistant to therapy. This resistance has been associated with the overexpression of *ABCB1*, which gives rise to the multidrug resistance (MDR) phenomenon. MDR is characterized by resistance to nonrelated drugs, and P-glycoprotein (encoded by *ABCB1*) has been implicated as the major cause of its emergence. Wnt signaling has been demonstrated to be important in several aspects of CML. Recently, Wnt signaling was linked to *ABCB1* regulation through its canonical pathway, which is mediated by β-catenin, in other types of cancer. In this study, we investigated the involvement of the Wnt/β-catenin pathway in the regulation of *ABCB1* transcription in CML, as the basal promoter of *ABCB1* has several β-catenin binding sites. β-catenin is the mediator of canonical Wnt signaling, which is important for CML progression.

**Methods:**

In this work we used the K562 cell line and its derived MDR-resistant cell line Lucena (K562/VCR) as CML study models. Real time PCR (RT-qPCR), electrophoretic mobility shift assay (EMSA), chromatin immunoprecipitation (ChIP), flow cytometry (FACS), western blot, immunofluorescence, RNA knockdown (siRNA) and Luciferase reporter approaches were used.

**Results:**

β-catenin was present in the protein complex on the basal promoter of *ABCB1* in both cell lines *in vitro*, but its binding was more pronounced in the resistant cell line *in vivo*. Lucena cells also exhibited higher β-catenin levels compared to its parental cell line. *Wnt1* and *β-catenin* depletion and overexpression of nuclear β-catenin, together with TCF binding sites activation demonstrated that *ABCB1* is positively regulated by the canonical pathway of Wnt signaling.

**Conclusions:**

These results suggest, for the first time, that the Wnt/β-catenin pathway regulates *ABCB1* in CML.

## Background

Chronic myeloid leukemia (CML) is a myeloproliferative disease characterized by the BCR-ABL constitutive tyrosine kinase (TK) oncoprotein, the result of the balanced reciprocal translocation of chromosomes 9 and 22 (t(9;22)(q34;q11)) [1]. BCR-ABL signaling is responsible for the pathogenesis of CML and is the primary molecular target for disease therapy with imatinib mesylate (Glivec, Gleevec, IM), a TK inhibitor. CML progresses in three phases: an initial phase known as the chronic phase (CP), the accelerated phase (AP) and the blastic crisis (BC) [2]. CML progression to BC has been associated, among others, with the canonical pathway of Wnt signaling. Activation of this pathway leads to nuclear accumulation of β-catenin, which activates the TCF/LEF1 family of transcriptional factors. The canonical pathway plays an important role in CML progression by activating several targets, such as c-MYC, ROK13A, cadherin, MDI1, prickle 1, and FZD2 [3]. Recently, this pathway was demonstrated to be crucial in disease maintenance through the sustenance of CML stem cells [4-6]. Hu and colleagues indicated that β-catenin is essential for the survival and self-renewal of CML stem cells even in mice subjected to kinase inhibition therapy [7].

Mechanisms surrounding the response to IM therapy in CML have been mostly associated with BCR-ABL oncoprotein mutations and *BCR-ABL* amplification. Nevertheless, some patients do not present either mechanism or respond to therapy, suggesting other mechanisms, the so-called BCR-ABL-independent mechanisms. Among them is the multidrug resistance (MDR) phenotype that is dependent on the expression of proteins that function as extrusive pumps [8-10]. In leukemia, the product of *ABCB1*, P-glycoprotein (Pgp), is most commonly implicated in the development of drug resistance. It is known that *ABCB1* can be regulated by several pathways in different conditions and that there is redundancy in this regulation [11]. However, despite the complex pattern of the *ABCB1* promoter region, the existence of seven TCF/LEF1 consensus binding sites on the basal promoter of this gene [12] indicates the possibility of *ABCB1* regulation by the canonical Wnt pathway. Indeed, Yamada and colleagues [13], Flahaut and colleagues [14] and Bourguignon and colleagues [15] revealed the involvement of the Wnt/β-catenin pathway in *ABCB1* regulation in early colorectal cancer, neuroblastoma and breast cancer, respectively. Regarding CML, studies with patients have shown that polymorphisms of the *ABCB1* gene can alter the response to therapy [16-21]. A few works have confirmed that *ABCB1* can be overexpressed in the AP of disease [22-26], and IM-resistant cell lines also overexpress *ABCB1*[27-29]. Our previously work have demonstrated that MDR cell line Lucena, which over-expresses *ABCB1* (800-fold increase) and Pgp (45-fold increase) is cross-resistant to IM and IM-resistant patients present *ABCB1* over-expressed, despite of disease phase [30]. Therefore, the aim of this work was to investigate the involvement of the WNT/β-catenin pathway in the regulation of *ABCB1* transcription in CML. Our results provide unprecedented information regarding *ABCB1* regulation in CML.

## Methods

### Culture conditions

Lucena (K562 multidrug-resistant cell line induced by vincristine (VCR)) cells overexpressing *ABCB1* were kindly provided by Dr^a^. Vivian Rumjanek (Departamento de Bioquímica Médica, Universidade Federal do Rio de Janeiro, Brazil) [31]. The human myelogenous leukemia cell line (K562) and its vincristine-resistant derivative, the Lucena cell line, were grown in RPMI 1640 medium (Invitrogen) supplemented with 10% FBS (Invitrogen), 50 units/mL penicillin G (Invitrogen), 50 μg/L streptomycin (Invitrogen) and 2 mM l-glutamine (Invitrogen) at 37 °C in a humidified atmosphere containing 5% CO_2_. Lucena medium was supplemented with 60 nM VCR (Sigma).

### Electrophoretic mobility shift assays (EMSAs)

Syntheses of double-stranded oligonucleotides for the seven TCF sites in the *ABCB1* sequence from the upstream promoter followed the protocols of Labialle and colleagues see reference [12]. They were named S1 to S7, with S7 being the shortest distance from the coding sequence. TCF sites are distributed throughout the *ABCB1* promoter, with S1 and S2 being distal sites.

S1: 5′-CAACTCG**TCAAAG**GAATTAT-3′

S2: 5′-GGTGTTGA**TCAAAG**GTACAA-3′

S3: 5′-GCAGAAC**TCAAAG**AAACAGA-3′

S4: 5′-ATGTCAAAA**CAAAG**GAGATT-3′

S5: 5′-AAA**CAAAG**TTTGCTCCTCTT-3′

S6: 5′-GTAGGAAATA**CAAAG**AATACT-3′

S7: 5′-GCCTAAGAA**CAAAG**AGAGAG-3′

Dephosphorylated oligonucleotides (25 pmol) were end-labeled with [γ-^32^P] ATP and T4 polynucleotide kinase. For binding reactions, 7 μg of nuclear protein extracts, prepared as previously described [32], was incubated with 80.000 cpm of labeled probe in 2 μL of 1× binding buffer (50 mM HEPES - pH 7.4, 300 mM KCl, 5 mM EDTA, 5 mM DTT, 11.5% Ficoll) and 1 μg of poly(dI-dC)(dI-dC) (GE) in a total volume of 20 μL for 40 min at room temperature (25 °C). Reactions were resolved by 4.5% polyacrylamide gel electrophoresis in 0.5× TBE for 90 min at 4 °C. In all EMSA experiments, the concentration chosen for competition experiments was a 200-fold molar excess. For competition reactions, a 200-fold molar excess of nonlabeled competitor DNA was added 20 min before the addition of the probe. The Opt oligonucleotide, described by Pizzatti and colleagues, was also used as a competitor because it only possesses the TCF consensus binding site in its sequence [33]. Opt 5′-GGTAAGA**TCAAAG**GG-3′

For supershift analysis of the *ABCB1* promoter, protein extracts were incubated for 2 h with 1 μg of the anti-β-catenin (Sigma) and anti-Smad8 (Santa Cruz Technologies) antibodies at 4 °C before the addition of the probe. Anti-Smad8 was used as a negative control.

### Chromatin immunoprecipitation (ChIP) assays on native chromatin

Chromatin from K562 and Lucena cells was fractionated by incubation of purified nuclei with micrococcal nuclease and its immunoprecipitation with anti-β-catenin antibody was performed as described previously [34]. DNA extractions from bound fractions were performed following the Abcam (www.abcam.com) protocol. The immunoprecipitated DNA was amplified for sequences containing binding sites by using the following *ABCB1* promoter sequence primers: *ABCB1*p (F) 5′-CAACTCGTCAAAGGAATTAT-3′ and *ABCB1*p (R) 5′-TTGTACCTTTGATCAACACC-3′.

Quantification was evaluated by RT-qPCR analysis. Immunoprecipitation of Protein A (Santa Cruz Technologies) was used for non-specific binding, and Smad8 (Santa Cruz Technologies) was used as a positive control.

### Real-time quantitative PCR (RT-qPCR)

Analysis of *ABCB1WNT1β-catenin* and *β-ACTIN* mRNA levels was performed by RT-qPCR. Two micrograms of Trizol (Invitrogen) extracted RNA from cell lines was treat with DNAse Amplification Grade I (Invitrogen) and reverse-transcribed with Superscript II Reverse Transcriptase® (Invitrogen). cDNAs dilutions (1:100) were mixed with SYBR Green PCR Master Mix® (Applied Biosystems) and the following primers: *ABCB1*: (F) 5′-CCC ATC ATT GCA ATA GCA GG-3′ and (R) 5′-GTT CAA ACT TCT GCT CCT GA-3′; *WNT1*: (F) 5′-TGG TTT GCA AAG ACC ACC TCC A-3′, and (R) 5′-TGA TTC CAG GAG GCA AAC GCA T-3′; *β-CATENIN*: (F) 5´-AAG ACA TCA CTG AGC CTG CCAT-3´ and (R) 5´-CGA TTT GCG GGA CAA AGG GCA A-3´; *β-ACTIN*: (F) 5′-ACC TGA GAA CTC CAC TAC CCT-3′ and (R) 5′-GGT CCC ACC CAT GTT CCA G-3′. RT-qPCR was performed in a Rotor Gene 6000 thermocycler (Corbett) with 50 cycles of 20 s at 95 °C, 30 s at 60 °C and 30 s at 72 °C. For each sample, the expression of target genes was normalized to *β-actin* mRNA levels. Changes in the mRNA levels of genes were evaluated [35].

### WNT/β-catenin activation by LiCl treatment

Cell cultures were exposed to 10 mM LiCl [36]. Treatment was performed in 12-well culture plates for 24 and 48 h at a cellular density of 2.0 × 10^5^ cells/mL. The toxicity of the assay was evaluated by FACS analysis, and *ABCB1* mRNA levels were analyzed by RT-qPCR.

### Flow cytometry (FACS) analysis

Viability was evaluated *via* the analysis of propidium iodide (PI) staining (Sigma-Aldrich). Briefly, K562 and Lucena cells (approximately 3.0 x 10^5^ cells) treated with 10 mM LiCl (24 h and 48 h) were harvested and washed in 500 μL of PBS. PI (1.5 μg/mL) was added to the incubated tubes prior to FACS analysis. PI(−) cells were considered viable. All procedures were performed according to the manufacturer’s protocol. For β-catenin expression detection, cells were harvested (approximately 2.5 x 10^5^ cells) and fixed with PBS/1% Formol. As follow, they were permeabilized with 0.5% Tween 20 to allow intracellular staining and labeled with anti-β-catenin polyclonal antibody (Sigma). Results are expressed as mean relative fluorescence intensity (MRFI), which was calculated by subtracting the mean fluorescence intensity (MFI) for specific antibody by the MFI of the respective secondary antibody (which served as negative isotype control). Ten thousand events were analyzed for each sample in a FACSCalibur Flow Cytometer (Becton Dickinson). The data were analyzed using CellQuest v.3.1 software (Becton Dickinson). All experiments were performed in triplicate.

### RNAi knockdown (siRNA) and transfection

All RNA oligonucleotides described in this study were synthesized and purified using high-performance liquid chromatography at Integrated DNA Technologies (Coralville), and the duplex sequences are available upon request. siRNA and transfections were performed following the manufacturer’s protocols of the TriFECTa Dicer-Substrate RNAi kit (Integrated DNA Technologies) and the Trifectin reagent (Integrated DNA Technologies). K562 and Lucena cells (5.5 × 10^4^ cells per well) were split in 24-well plates at 60% confluence in RPMI medium 1 day prior to transfection. The TriFECTa kit contains control sequences for RNAi experiments, including a fluorescently labeled transfection control duplex and a scrambled universal negative control RNA duplex that is absent in human, mouse and rat genomes. Fluorescence microscopy was used to monitor the transfection efficiency according to the manufacturer’s recommendations. Only experiments in which transfection efficiencies were ≥ 80% were evaluated. mRNA levels were measured 48 h after transfection. Duplexes were evaluated at 10 nM. All transfections were minimally performed in duplicate, and the data were averaged. *Wnt1* and *β-Catenin* depletion and RT-qPCR analyses were performed as described above.

### Western blot analysis

Cell lysates from K562 and Lucena cells (control - untreated - and treated with LiCl 10 mM) were run on 15% sodium dodecyl sulfate-polyacrylamide gels (SDS-PAGE), transferred to nitrocellulose membranes (Bioead) and incubated with β-catenin (Santa Cruz Technologies), P-GSK3α/β (Cell signaling) and α-tubulin (Sigma) antibodies. Antibody binding was detected using enhanced chemiluminescence ECL Plus Western Blotting detection Reagents (GE).

### Immunofluorescence staining and confocal laser microscopy

Cytospin preparations of K562 and Lucena cells were fixed with methanol, permeabilized with 0.2% triton X-100 PBS, and incubated for 1 h at room temperature with 1% bovine serum albumin (BSA) and 2.0% FBS blocking buffer under shaking. Slides were rinsed once with PBS and 0.05% Tween 20 and then incubated with appropriately diluted primary antibodies in PBS. Cells were incubated with anti-β-catenin polyclonal antibody (Sigma) overnight at 4 °C. After incubation, the slides were rinsed three times and incubated with Alexa® 546 conjugated anti-rabbit IgG (Molecular Probes) for 1 h at room temperature. Sections from each sample were incubated with secondary antibody and served as negative isotype control. The slides were rinsed three times, air-dried and mounted in an antifading medium containing 4',6-diamidino-2-phenylindole (DAPI) (Vector Labs). Expression and localization of the proteins were observed with a Leica TCS-SP5 AOBS confocal laser scanning microscope (Leica), for capturing representative images of each sample.

### Reporter vectors design

The *ABCB1* reporter constructs were synthesized by Gene Art (Germany) and cloned into the firefly pGL3-Basic vector (Promega) upstream of the Luciferase reporter gene. The constructs named pGL3α, containing just the basal promoter (−1019/+1); pGL3β, containing the basal promoter and one TCF binding site (−1067/+1) and pGL3γ, containing the basal promoter and three TCF binding sites (−3187/+1) were inserted into KpnI and BgLII restriction sites of pGL3-basic.

### Transient transfection and luciferase reporter assay

For the transient assays, 1.0 x 10^5^ cells from both cell lines (with or without LiCl 10 mM treatment) were co-transfected using Lipofectamine LTX 2000 (Invitrogen) with 1 *μ*g of each Luciferase construct and 100 ng of pRL-SV40 vector (Promega), according to the manufacturer’s instructions. Firefly and Renilla Luciferase activities were measured in cell lysates 48 hours after transfection using the DualGlo Luciferase Assay System (Promega) on a Veritas TM Microplate Luminometer (Turner Biosystems), following the manufacturer’s protocol. All experiments were performed in triplicate. Ratios of Renilla luciferase readings to firefly luciferase readings were taken for each experiment and triplicates were averaged. The average values of the tested constructs were normalized to the activity of the empty pGL3-basic vector, which was arbitrarily set at value 1.

### Statistical analysis

Comparison between K562 and Lucena results from different assays was performed by an unpaired *t*-test. *P*- Values less than 0.05 were considered as statistically significant (*p < 0.05,**p < 0.01, and ***p < 0.001). Statistical analyses and graphical representations were performed using GraphPad Prism™ software (GraphPad).

## Results

### β-catenin binds to the *ABCB1* promoter at the TCF-binding site

**Figure 1:**
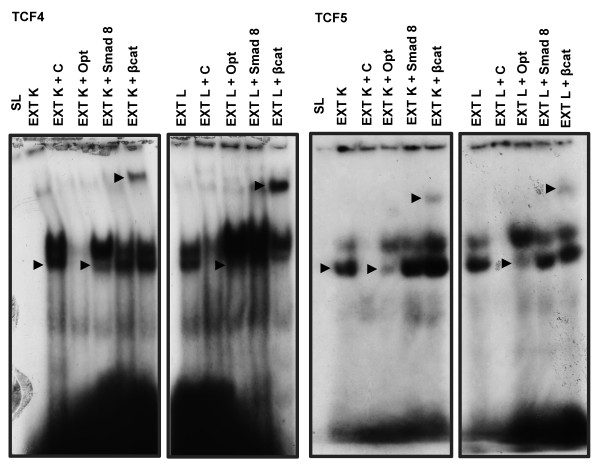
**EMSA using oligonucleotides of different TCF consensus binding sites in the*ABCB1*promoter and protein extracts from K562 and Lucena cells.** EMSAs to verify specific binding using the S4 and S5 oligonucleotides and protein extracts from K562 and Lucena cells. The specificity of the DNA-protein complex is demonstrated by competition reactions with 200-fold excess unlabeled oligonucleotide and by supershift assays. SL – Migration of the probe alone. “+C”- Competition reactions with 200-fold excess unlabeled probe. “+Opt” – Competition reactions with 200-fold excess unlabeled wild-type oligonucleotide for the TCF consensus binding site. EXT K – protein extract from K562 cells. EXT L – protein extract from Lucena cells. “+Smad 8”– Supershift reactions using Smad8 antibody. . “+ βcat” – Supershift reactions using β-catenin antibody. The arrows show specific binding.

Protein binding to the seven oligonucleotides containing TCF consensus binding sites (S1, S2, S3, S4, S5, S6 and S7) was determined by EMSA (see additional file 1 for all EMSAs for TCF binding sites). We also performed competition assays to verify whether protein complex binding was specific for the TCF consensus binding site. Competition analyses using a 200-fold excess of unlabeled S4, S5 and Opt oligonucleotides demonstrated that the specific binding was reduced (Figure1). In addition, we investigated the presence of β-catenin in the protein complexes formed at the TCF consensus binding site in both S4 and S5 oligonucleotides as these TCF binding sites showed the clearer and stronger signal in EMSA analysis. Supershift assays were performed using human β-catenin antibody, and these assays demonstrated that β-catenin was present in the protein complexes (Figure1). These results suggest that the TCF consensus binding site is important for the formation of a protein complex. The appearance of a shifted band in the supershift assay with both the S4 and S5 oligonucleotides ensures the presence of β-catenin among protein complexes binding at the *ABCB1* promoter.

**Figure 2:**
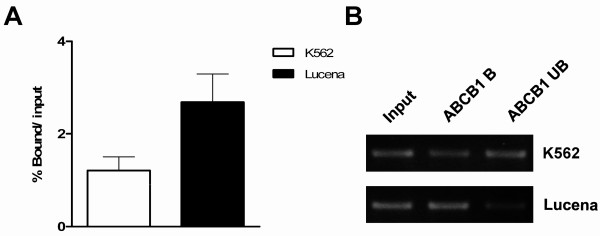
**ChIP assay for*in vivo*quantification of β-catenin binding to the*ABCB1*promoter.** (**A**) RT-qPCR quantification of β-catenin binding in K562 and Lucena cells. DNA amplification was quantified in bound and unbound fractions after normalization with protein A unspecific amplification. Normalized fractions were used to calculate the bound/input ratio. (**B**) Representative agarose gel - qualitative analysis – of *ABCB1* promoter amplification for β-catenin ChIP assay. Input: bound and unbound fractions; B: bound; UB: unbound.

To confirm the EMSA results, we performed ChIP assays. The ChIP assay allows *in vivo* analysis of nuclear protein-DNA interactions. Chromatin fractions bound to the β-catenin antibody in K562 and Lucena cells were quantified by RT-qPCR using primers to amplify the promoter region that contains TCF binding sites. A 2-fold increase in β-catenin binding was verified in Lucena cells compared to that in K562 cells after normalization with unspecific binding of protein A (Figure2A). Qualitative analysis of *ABCB1* promoter amplification is shown in Figure2B.

**Figure 3:**
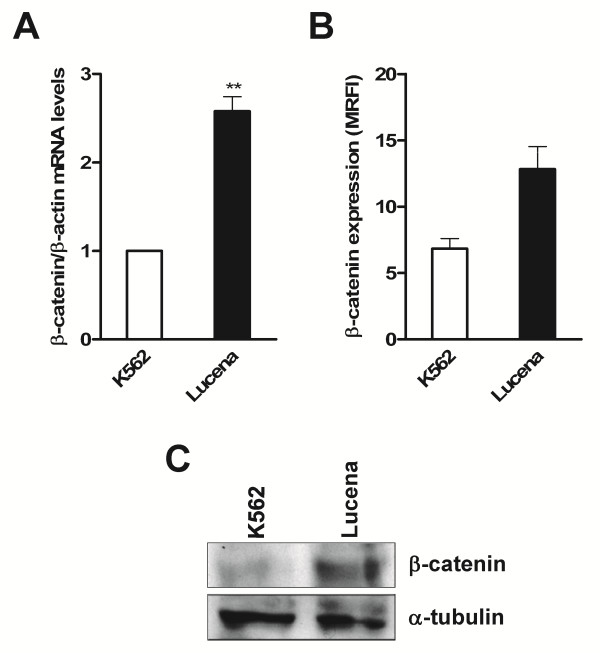
**β-catenin expression levels in K562 and Lucena cells.** (**A**) RT-qPCR analysis of *β-catenin* mRNA levels. Raw expression values were normalized to β-actin expression. (**B**) β-catenin expression by FACS, represented as MRFI. Secondary antibody was used as isotype antibody control. (**C**) Representative western blot analysis of β-catenin expression. 50 μg of protein extracts from both cell lines were separated SDS-PAGE and probed with anti- β-catenin antibody. α-tubulin was used for constitutive expression. Values represent the means of three independent determinations ± s.d.

**Figure 4:**
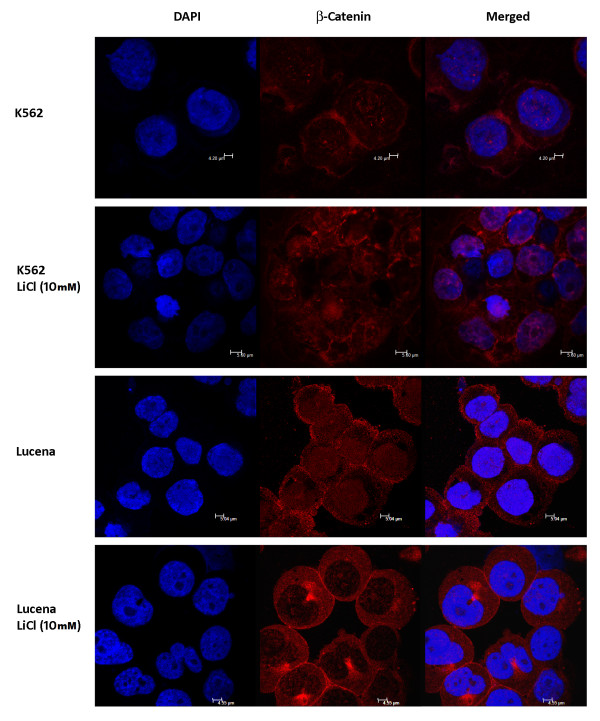
**Distribution of β-catenin in K562 and Lucena cells.** Confocal microscopy of cytospin preparations showing the relative nuclear, membrane and cytoplasmic distribution of β-catenin in K562 and Lucena cells exposed to LiCl 10 mM treatment for 24 h. Nuclei β-catenin density significantly increases upon treatment, compared to untreated cells. Nuclei are stained with DAPI (blue). Micrograph panel is representative of three experiments for each condition.

In order to investigate if the more pronounced bind observed in ChIP assay was due to different β-catenin expression between cell lines, we evaluated its expression by RT-qPCR, western blot, flow cytometry and immunofluorescence assays (Figure3 and Figure4). The results show a higher expression of β-catenin in Lucena cell line.

### The Wnt/β-catenin signaling pathway regulates *ABCB1* expression

**Figure 5:**
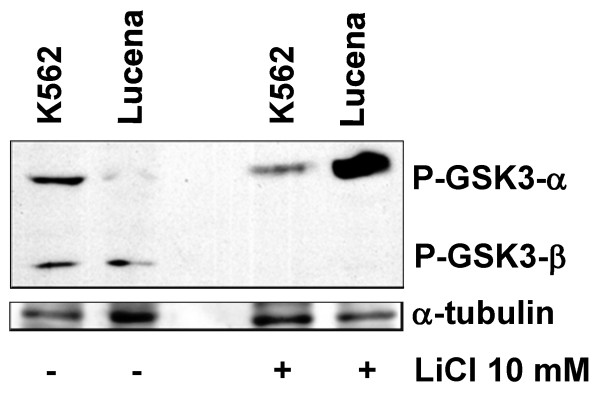
**Western Blot analysis of GSK3 activation.** Representative western blot analysis of P-GSK3 from both cell lines protein extracts, with or without LiCl 10 mM treatment. 50 μg of protein extracts were separated SDS-PAGE and probed with anti-P-GSK3 antibody. α-tubulin was used for constitutive expression. For each treatment results, representative of three independent experiments are shown.

To verify whether the binding of β-catenin to the *ABCB1* promoter could lead to *ABCB1* transcriptional activation, we activated the canonical WNT pathway in K562 and Lucena cells by LiCl 10 mM treatment, as described by Stambolic and colleagues see reference [36]. LiCl inhibits GSK3-β, resulting in β-catenin stabilization and its consequent nuclear translocation. This treatment is currently used for this purpose in the literature [37]. The nuclear translocation of β-catenin was more pronounced in K562 cell line than in Lucena cell line (Figure4). Moreover after LiCl treatment we demonstrated that phosphorylated GSK3-β was abolished in both cell lines (Figure5) indicating that degradation of β-catenin was prevented, enhancing its nuclear translocation (Figure5).

**Figure 6:**
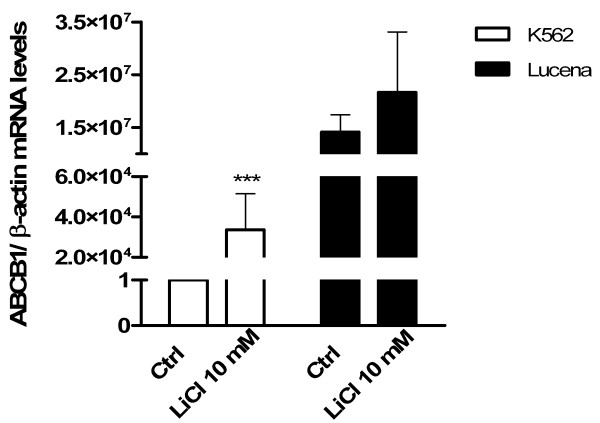
**Wnt/β-catenin pathway activation increases*ABCB1*expression.** Increases in *ABCB1* mRNA levels were evaluated after LiCl 10 mM treatment for 24 h. Total RNA was isolated and used in RT-qPCR to determine changes in *ABCB1* mRNA levels after normalization to *β-actin* expression. Values represent the means of three independent determinations ± s.d.

We examined cell viability by FACS analysis prior to RNA extraction (data not shown). As LiCl treatment did not alter cell viability, we evaluated *ABCB1* mRNA levels in K562 and Lucena cells treated with LiCl 10 mM by RT-qPCR analysis at 24 h. Untreated cells were used as a control. The increase in *ABCB1* mRNA levels was more significant in K562 cells than in Lucena cells (Figure6).

**Figure 7:**
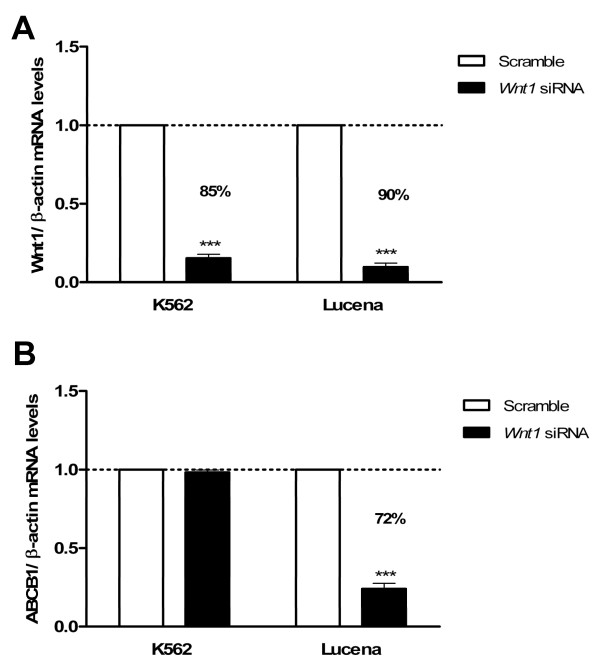
**Expression of*ABCB1*mRNA levels after*Wnt1*depletion in CML cell lines.** (**A**) *WNT1* siRNA in K562 and Lucena cells. (**B**) Analysis of *ABCB1* mRNA levels after *Wnt1* depletion. Total RNA was isolated and used in RT-qPCR analysis to determine changes in *ABCB1* mRNA levels after normalization to *β-actin* expression. All data were presented as fold inductions relative to control group expression (scrambled). Values represent the means of three independent determinations ± s.d.

**Figure 8:**
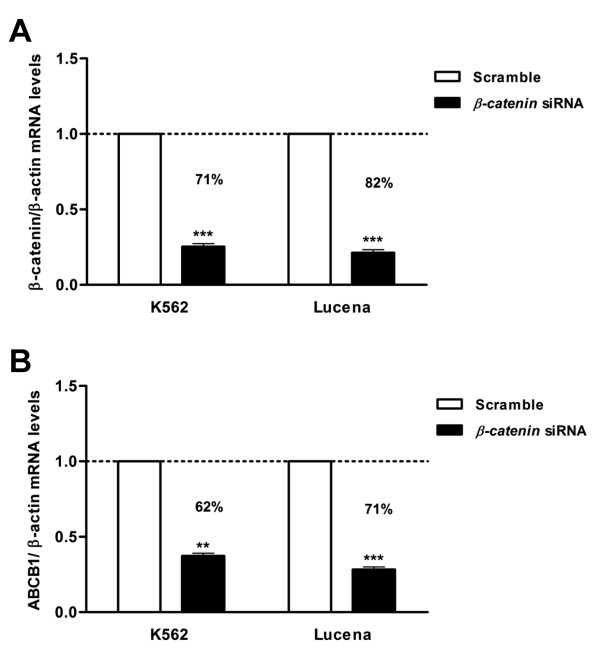
**Expression of*ABCB1*mRNA levels after*β-catenin*depletion in CML cell lines.** (**A**) *β-catenin* siRNA in K562 and Lucena cells. (**B**) Analysis of *ABCB1* mRNA levels after *β-catenin* depletion. Total RNA was isolated and used in RT-qPCR analysis to determine changes in *ABCB1* mRNA levels after normalization to *β-actin* expression. All data were presented as fold inductions relative to control group expression (scrambled). Values represent the means of three independent determinations ± s.d.

To strengthen our observations, we performed functional analyses of *WNT1* and *β-catenin* depletion in K562 and Lucena cell lines using RT-qPCR. Using a siRNA approach after 48 h of transfection, a reduction in *WNT1* expression of more than 85% and *β-catenin* of more than70% were achieved in both cell lines when compared to scrambled control sequence-treated cells (Figure7A and 8A). Thus, we used these samples to evaluate *ABCB1* mRNA levels to verify how the Wnt pathway could be involved in *ABCB1* regulation. *WNT1* depletion in Lucena cells resulted in an 80% reduction in *ABCB1* expression (Figure7B). Despite the 85% reduction of *WNT1* expression in siRNA-treated K562 cells, we did not find evidence of significant reductions in *ABCB1* mRNA levels compared to the levels in scramble control-treated cells, suggesting that probably other Wnt ligands could play a role in this activation (Figure7B).

However *β-catenin* reduction in both cell lines confirmed that indeed the canonical WNT pathway regulates *ABCB1* expression as shown by 60% and 71% reduction of *ABCB1* mRNA levels in K562 and Lucena cells respectively (Figure8B).

### *ABCB1* promoter TCF binding sites role in transcriptional activity

**Figure 9:**
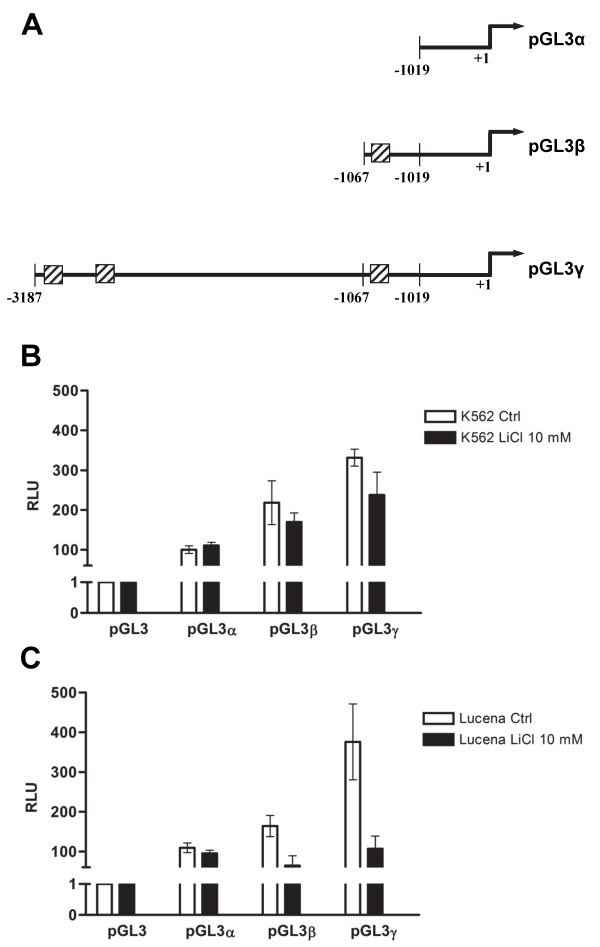
***ABCB1*promoter TCF binding sites transcription activity using reporter plasmid pGL3 basic.** (**A**) Scheme of constructs with TCF binding sites. (**B**) Luciferase activity reporter assay in K562 cells with or without LiCl 10 mM for 24 h. (**C**) Luciferase activity reporter assay in Lucena cells with or without LiCl 10 mM for 24 h. All luciferase assay results expressed as relative light units (RLU).

To further evaluate the relative contribution of TCF transcription factor to the regulation of *ABCB1* promoter activity, we performed transient transfection assays using K562 and Lucena cells with constructions containing TCF binding sites (Figure9A). These constructions were transfected with or without LiCl 10 mM and Luciferase activity was measured using Luciferase assay approach.

These results showed that Luciferase activity increases in the presence of TCF binding site in both K562 and Lucena cell lines (Figure9B, 9C). Even with only one TCF binding site-pGL3β construct transfection, we observed a higher Luciferase activity compared with basal promoter without TCF binding sites. The Luciferase activity increases with constructions with more than one TCF binding sites (Figure9B, 9C).

In K562 and Lucena cells treated with LiCl we observed a reduction in the Luciferase activity when compared with the untreated cells. As LiCl treatment results in the translocation of β-catenin to the nucleus this reduction reflects the lack of cytoplasmatic β-catenin necessary to activate TCF binding sites in the constructs that are in the cytoplasm.

## Discussion

Resistance to chemotherapy is a recurrent issue in all cancer types. Because CML has the propensity to evolve from the CP to the AP and BC, with different responses to targeted therapy such as TK inhibitors, the molecular understanding of the mechanism of resistance in this neoplasia is advancing rapidly.

IM was the first molecularly targeted therapy rationally designed to specifically inhibit BCR-ABL TK activity [38]. However, despite the effectiveness and good tolerability of IM, drug resistance does emerge. Although a hematological response is observed in over 95% of CP patients, primary resistant can occur [39]. Otherwise, AP patients initially respond to IM but inevitably relapse with treatment-refractory disease because they acquire other mutations in addition to BCR-ABL amplification or kinase domain mutations [40-42].

Since the demonstration that IM could be extruded from CML cells through Pgp action [43,44], *ABCB1* has become an interesting subject in IM resistance studies. Our previous results indicated that *ABCB1* is overexpressed in CML patients with intrinsic and acquired resistance to IM therapy compared to its expression in IM-responsive patients and healthy bone marrow donors see reference [30]. This finding contrasts the idea that only individuals in the BC stage can exhibit *ABCB1* overexpression, as suggested in the literature. Even though we have analyzed a small cohort of patients, our results corroborate those of Vasconcelos and colleagues see reference [24], ratifying the importance of *ABCB1*/Pgp in CML.

Interestingly, in CML, Jamieson and colleagues demonstrated that the granulocyte-macrophage progenitor pools from patients in BC and IM-resistant patients exhibited elevated levels of nuclear β-catenin compared with those in granulocyte-macrophage progenitors from healthy donors. Moreover, these progenitors acquired self-renewal ability [45]. These data indicated the important role of the Wnt/β-catenin pathway in the self-renewal of CML progenitors and the acquisition of resistance. Wnt signaling involvement in TK inhibitor resistance was also demonstrated through its noncanonical pathway by Gregory and colleagues [46]. Their results indicated that Wnt/Ca^2+^/NFAT signaling maintains the survival of Ph^+^ leukemic cells under BCR-ABL inhibition. Altogether, we can speculate that the deregulation of Wnt signaling leads to key modifications in the biology of cells, allowing them to become intrinsically more resistant to drug therapy. However, a link between TK inhibitor resistance, Wnt signaling and drug efflux mechanisms such as MDR has never been considered. Despite some recent findings demonstrating that TCF consensus sites for β-catenin were functional in the *ABCB1* promoter in other types of cancer, in CML, this regulation has not yet been investigated.

In this work, using MDR (overexpressing *ABCB1*) and non-MDR cell lines (Lucena and K562, respectively) as models of CML, we demonstrated through EMSA and ChIP analyses that β-catenin binds to the TCF/LEF consensus binding site in the *ABCB1* promoter. RT-qPCR analyses indicated that this binding occurred at 2-fold higher levels in Lucena cells than in K562 cells. As it has been demonstrated that the BCR-ABL protein can establish β-catenin expression in CML *via* TK-mediated phosphorylation [47], this finding suggests that in drug resistance in CML, the canonical Wnt pathway could be more strongly activated to positively regulate *ABCB1* transcription, as previously evidenced in other types of cancer. Interestingly we could demonstrate by RT-qPCR, western blot and FACS, β-catenin higher expression in the MDR cell line. Furthermore, it is known that BCR is a negative regulator of the Wnt/β-catenin pathway. The fusion gene *BCR-ABL* formed in CML decreases BCR transcription and BCR translation, and thus, there is no more BCR available to complex with β-catenin, leading to the translocation of β-catenin to the nucleus and Wnt/β-catenin pathway activation [48].

To verify whether Wnt/β-catenin could regulate *ABCB1* mRNA levels, we modulated the canonical pathway in CML cell lines. We demonstrated that activation of β-catenin signaling by LiCl treatment resulted in increased *ABCB1* mRNA levels in both cell lines, with higher levels observed in K562 cells. As discussed previously, K562 cells exhibited less β-catenin binding to the *ABCB1* promoter than Lucena cells. *ABCB1* mRNA levels were not altered significantly in Lucena cells, suggesting that these cells exhibit saturation of the Wnt pathway. By silencing the pathway using a siRNA approach with *WNT 1* and *β-catenin* knockdown, we verified the opposite—a significant decrease in *ABCB1* mRNA levels in Lucena cells—indicating that when Wnt/β-catenin signaling is downregulated, *ABCB1* transcription is also downregulated.

*ABCB1* gene promoter presents seven TCF binding sites that have been until now poorly investigated in the regulation of gene transcriptional activity in cancer cells. However the more proximal TCF binding site have already been demonstrated to be functional in some cancer types see references [13-15]. In this study we could demonstrate that all the seven TCF binding sites are potentially functional (supplementary material) and moreover we showed that two of them are functional *in vivo*. Transfection experiments suggest that TCF binding sites can function in combination to enhance transcriptional activity.

These findings suggest that the canonical pathway of Wnt signaling regulates *ABCB1* in CML. Several studies have demonstrated that quiescent CML stem cells do not undergo apoptosis even in the presence of high-dose or more potent TK inhibitors. Moreover, seminal studies demonstrated that a quiescent population of CML stem cells with BCR-ABL kinase domain mutation that is detectable before the initiation of IM therapy gives rise to leukemic cells that persist after treatment see references [1-7,49-52]. These findings suggest that CML stem cells contribute to CML persistence and disease progression. A question to be further addressed is whether *ABCB1* is also regulated by the canonical Wnt pathway in CML stem cells, as this pathway has also been correlated with self-renewal.

CML stem cells and normal hematopoietic stem cells (HSC) share several characteristics despite exhibiting remarkable differences. Self-renewal is an essential stem cell property, but self-renewal pathway activation has also been increasingly recognized as a hallmark of cancer. Interestingly, HSC and CML stem cells also exhibit increased levels of drug efflux-related molecules such as the product of *ABCB1*, Pgp and decreased levels of OCT1, a transporter involved in the uptake of IM, rendering them more resistant to drugs [53,54].

## Conclusion

By the data presented in this work, we provided evidence that the canonical pathway of Wnt signalling is involved in *ABCB1* transcriptional activation in CML.

## Competing interests

The authors declare that they have no competing interests.

## Authors’ contributions

SC performed the experiments, statistical analysis and drafted the manuscript. RB participated and assisted the electrophoretic mobility shift assays and Luciferase experiments. BR participated in flow cytometry experiments. MTLCB performed immunofluorescence assays. LP and EA made substantial contributions to the study conception and design and critically revised the manuscript for intellectual content. All authors read and approved the final manuscript.

## References

[B1] Jiang X Zhao Y Smith C Gasparetto M Turhan A Eaves A Eaves C Chronic myeloid leukemia stem cells possess multiple unique features of resistance to BCR-ABL targeted therapies Leukemia 2007 21 926 935 17330101 10.1038/sj.leu.2404609

[B2] Jørgensen HG Holyoake TL Characterization of cancer stem cells in chronic myeloid leukaemia Biochem Soc Trans 2007 35 1347 1351 17956348 10.1042/BST0351347

[B3] Radich JP Dai H Mao M Oehler V Schelter J Druker B Sawyers C Shah N Stock W Willman CL Friend S Linsley PS Gene expression changes associated with progression and response in chronic myeloid leukemia Proc Natl Acad Sci U S A 2006 103 2794 2799 16477019 10.1073/pnas.0510423103 PMC1413797

[B4] Zhao C Blum J Chen A Kwon HY Jung SH Cook JM Lagoo A Reya T Loss of beta-catenin impairs the renewal of normal and CML stem cells in vivo Cancer Cell 2007 12 528 541 18068630 10.1016/j.ccr.2007.11.003 PMC2262869

[B5] Chen Y Peng C Sullivan C Li D Li S Critical molecular pathways in cancer stem cells of chronic myeloid leukemia Leukemia 2010 24 1545 1554 20574455 10.1038/leu.2010.143 PMC3130198

[B6] Deshpande AJ Buske C Knocking the Wnt out of the sails of leukemia stem cell development Cell Stem Cell 2007 1 597 598 18371398 10.1016/j.stem.2007.11.006

[B7] Hu Y Chen Y Douglas L Li S beta-Catenin is essential for survival of leukemic stem cells insensitive to kinase inhibition in mice with BCR-ABL-induced chronic myeloid leukemia Leukemia 2009 23 109 116 18818703 10.1038/leu.2008.262

[B8] Hochhaus A Chronic myelogenous leukemia (CML): resistance to tyrosine kinase inhibitors Ann Oncol 2006 17 274 279 10.1093/annonc/mdl273 17018738

[B9] Mughal TI Goldman JM Emerging strategies for the treatment of mutant Bcr-Abl T315I myeloid leukemia Clin Lymphoma Myeloma 2007 7 S81 S84 17382017 10.3816/clm.2007.s.006

[B10] Quintás-Cardama A Kantarjian HM Cortes JE Mechanisms of Primary and Secondary Resistance to Imatinib in Chronic Myeloid Leukemia Cancer Control 2009 16 122 131 19337198 10.1177/107327480901600204

[B11] Shtil AA Azare J Redundancy of biological regulation as the basis of emergence of multidrug resistance Int Rev Cytol 2005 246 1 29 16164965 10.1016/S0074-7696(05)46001-5

[B12] Labialle S Gayet L Marthinet E Rigal D Baggetto LG Transcriptional regulators of the human multidrug resistance 1 gene: recent views Biochem Pharmacol 2002 64 943 948 12213590 10.1016/s0006-2952(02)01156-5

[B13] Yamada T Takaoka AS Naishiro Y Hayashi R Maruyama K Maesawa C Ochiai A Hirohashi S Transactivation of the multidrug resistance 1 gene by T-cell factor 4/beta-catenin complex in early colorectal carcinogenesis Cancer Res 2000 60 4761 4766 10987283

[B14] Flahaut M Meier R Coulon A Nardou KA Niggli FK Martinet D Beckmann JS Joseph JM Mühlethaler-Mottet A Gross N The Wnt receptor FZD1 mediates chemoresistance in neuroblastoma through activation of the Wnt/beta-catenin pathway Oncogene 2009 28 2245 2256 19421142 10.1038/onc.2009.80

[B15] Bourguignon LYW Xia W Wong G Hyaluronan-mediated CD44 interaction with p300 and SIRT1 regulates beta-catenin signaling and NFkappaB-specific transcription activity leading to MDR1 and Bcl-xL gene expression and chemoresistance in breast tumor cells J Biol Chem 2009 284 2657 2671 19047049 10.1074/jbc.M806708200 PMC2631959

[B16] Gurney H Wong M Balleine RL Rivory LP McLachlan AJ Hoskins JM Wilcken N Imatinib disposition and ABCB1 (MDR1, P-glycoprotein) genotype Clin Pharmacol Ther 2007 82 33 40 17495881 10.1038/sj.clpt.6100201

[B17] Dulucq S Bouchet S Turcq B Lippert E Etienne G Reiffers J Molimard M Krajinovic M Mahon FX Multidrug resistance gene (MDR1) polymorphisms are associated with major molecular responses to standard-dose imatinib in chronic myeloid leukemia Blood 2008 112 2024 2027 18524988 10.1182/blood-2008-03-147744

[B18] Kim DH Sriharsha L Xu W Kamel-Reid S Liu X Siminovitch K Messner HA Lipton JH Clinical relevance of a pharmacogenetic approach using multiple candidate genes to predict response and resistance to imatinib therapy in chronic myeloid leukemia Clin Cancer Res 2009 15 4750 4758 19584153 10.1158/1078-0432.CCR-09-0145

[B19] Ni LN Li JY Miao KR Qiao C Zhang SJ Qiu HR Qian SX Multidrug resistance gene (MDR1) polymorphisms correlate with imatinib response in chronic myeloid leukemia Med Oncol 2011 28 265 269 20204543 10.1007/s12032-010-9456-9

[B20] Maffiolia M Camósa M Gayaa A Hernández-Boludab JC Álvarez-Larránc A Domingoa A Granella M Guillemb V Vallansota R Costad D Bellosillo B Colomerd D Cervantesa F Correlation between genetic polymorphisms of the hOCT1 and MDR1 genes and the response to imatinib in patients newly diagnosed with chronic-phase chronic myeloid leukemia Leuk Res 2011 35 1014 1019 21185600 10.1016/j.leukres.2010.12.004

[B21] Yamakawa Y Hamada A Nakashima R Yuki M Hirayama C Kawaguchi T Saito H Association of Genetic Polymorphisms in the Influx Transporter SLCO1B3 and the Efflux Transporter ABCB1 With Imatinib Pharmacokinetics in Patients With Chronic Myeloid Leukemia Ther Drug Monit 2011 33 244 250 21311410 10.1097/FTD.0b013e31820beb02

[B22] List AF Kopecky KJ Willman CL Head DR Slovak ML Douer D Dakhil SR Appelbaum FR Cyclosporine inhibition of P-glycoprotein in chronic myeloid leukemia blast phase Blood 2002 100 1910 1912 12176916

[B23] Stromskaya TP Rybalkina EY Kruglov SS Zabotina TN Mechetner EB Turkina AG Stavrovskaya AA Role of P-glycoprotein in evolution of populations of chronic myeloid leukemia cells treated with imatinib Biochemistry (Mosc) 2008 73 29 37 18294126 10.1134/s0006297908010045

[B24] Vasconcelos FC Silva KL Souza PS Silva LF Moellmann-Coelho A Klumb CE Maia RC Variation of MDR proteins expression and activity levels according to clinical status and evolution of CML patients Cytometry B Clin Cytom 2011 80 158 166 21520403 10.1002/cyto.b.20580

[B25] Racil Z Razga F Polakova KM Buresova L Polivkova V Dvorakova D Zackova D Klamova H Cetkovsky P Mayer J Assessment of adenosine triphosphate-binding cassette subfamily B member 1 (ABCB1) mRNA expression in patients with de novo chronic myelogenous leukemia: the role of different cell types Leuk Lymphoma 2011 52 331 334 21133723 10.3109/10428194.2010.533220

[B26] Reis FR Vasconcelos FC Pereira DL Moellman-Coelho A Silva KL Maia RC Survivin and P-glycoprotein are associated and highly expressed in late phase chronic myeloid leukemia Oncol Rep 2011 26 471 478 21567097 10.3892/or.2011.1296

[B27] Mahon FX Deininger M Schultheis B Chabrol J Reiffers J Goldman JM Melo JV Selection and characterization of BCR-ABL positive cell lines with differential sensitivity to the signal transduction inhibitor STI571: diverse mechanisms of resistance Blood 2000 96 1070 1079 10910924

[B28] Tang C Schafranek L Watkins DB Parker WT Moore S Prime JA White DL Hughes TP Tyrosine kinase inhibitor resistance in chronic myeloid leukemia cell lines: investigating resistance pathways Leuk Lymphoma 2011 52 2139 2147 21718141 10.3109/10428194.2011.591013

[B29] Yamada O Ozaki K Furukawa T Machida M Wang YH Motoji T Mitsuishi T Akiyama M Yamada H Kawauchi K Matsuoka R Activation of STAT5 confers imatinib resistance on leukemic cells through the transcription of TERT and MDR1 Cell Signal 2011 23 1119 1127 21356308 10.1016/j.cellsig.2011.02.005

[B30] Corrêa S Pizzatti L Du Rocher B Mencalha A Pinto D Abdelhay E A comparative proteomic study identified LRPPRC and MCM7 as putative actors in imatinib mesylate cross-resistance in Lucena cell line Proteome Sci 2012 10 23 Epub ahead of print 22458888 10.1186/1477-5956-10-23 PMC3361502

[B31] Rumjanek VM Trindade GS Wagner-Souza K de-Oliveira MC Marques-Santos LF Maia RC Capella MA Multidrug resistance in tumour cells: characterization of the multidrug resistant cell line K562-Lucena 1 An Acad Bras Cienc 2001 73 57 69 11246270 10.1590/s0001-37652001000100007

[B32] Binato R Alvarez Martinez CE Pizzatti L Robert B Abdelhay E SMAD 8 binding to mice Msx1 basal promoter is required for transcriptional activation Biochem J 2006 393 141 150 16101586 10.1042/BJ20050327 PMC1383672

[B33] Pizzatti L Binato R Cofre J Gomes BE Dobbin J Haussmann ME D'Azambuja D Bouzas LF Abdelhay E SUZ12 is a candidate target of the non-canonical WNT pathway in the progression of chronic myeloid leukemia Genes Chromosomes Cancer 2010 49 107 118 19847889 10.1002/gcc.20722

[B34] Wagschal A Delaval K Pannetier M Arnaud P Feil R Chromatin Immunoprecipitation (ChIP) on Unfixed Chromatin from Cells and Tissues to Analyze Histone Modifications Cold Spring Harb. Protoc 2007 10.1101/pdb.prot4767 21357105

[B35] Livak KJ Schmittgen TD Analysis of relative gene expression data using real-time quantitative PCR and the 2(−Delta Delta C (T)) Method Methods 2001 25 402 408 11846609 10.1006/meth.2001.1262

[B36] Stambolic V Ruel L Woodgett JR Lithium inhibits glycogen synthase kinase-3 activity and mimics wingless signalling in intact cells Curr Biol 1996 6 1664 1668 8994831 10.1016/s0960-9822(02)70790-2

[B37] Lim JC Kania KD Wijesuriya H Chawla S Sethi JK Pulaski L Romero IA Couraud PO Weksler BB Hladky SB Barrand MA Activation of beta-catenin signalling by GSK-3 inhibition increases p-glycoprotein expression in brain endothelial cells J Neurochem 2008 106 1855 1865 18624906 10.1111/j.1471-4159.2008.05537.x PMC4303914

[B38] Melo JV Barnes DJ Chronic myeloid leukaemia as a model of disease evolution in human cancer Nat Rev Cancer 2007 7 441 453 17522713 10.1038/nrc2147

[B39] Perrotti D Jamieson C Goldman J Skorski T Chronic myeloid leukemia: mechanisms of blastic transformation J Clin Invest 2010 120 2254 2264 20592475 10.1172/JCI41246 PMC2898591

[B40] Jabbour E Fava C Kantarjian H Advances in the biology and therapy of patients with chronic myeloid leukaemia Best Prac Res Clin Haematol 2009 22 395 407 10.1016/j.beha.2009.09.002 19959090

[B41] Swords R Alvarado Y Giles F Novel Abl kinase inhibitors in chronic myeloid leukemia in blastic phase and Philadelphia chromosome-positive acute lymphoblastic leukemia Clin Lymphoma Myeloma 2007 7 S113 S119 17382020 10.3816/clm.2007.s.011

[B42] Roychowdhury S Talpaz M Managing resistance in chronic myeloid leukemia Blood Reviews 2011 25 279 290 21982419 10.1016/j.blre.2011.09.001

[B43] Hegedus T Orfi L Seprodi A Váradi A Sarkadi B Kéri G Interaction of tyrosine kinase inhibitors with the human multidrug transporter proteins, MDR1 and MRP1 Biochim Biophys Acta 2002 1587 318 325 12084474 10.1016/s0925-4439(02)00095-9

[B44] Hamada A Miyano H Watanabe H Saito H Interaction of Imatinib Mesilate with Human P-Glycoprotein J Pharmacol Exp Ther 2003 307 824 828 12975485 10.1124/jpet.103.055574

[B45] Mahon FX Belloc F Lagarde V Chollet C Moreau-Gaudry F Reiffers J Goldman JM Melo JV MDR1 gene overexpression confers resistance to imatinib mesylate in leukemia cell line models Blood 2003 101 2368 2373 12609962 10.1182/blood.V101.6.2368

[B46] Jamieson CH Ailles LE Dylla SJ Muijtjens M Jones C Zehnder JL Gotlib J Li K Manz MG Keating A Sawyers CL Weissman IL Granulocyte-macrophage progenitors as candidate leukemic stem cells in blast-crisis CML N Engl J Med 2004 351 657 667 15306667 10.1056/NEJMoa040258

[B47] Gregory MA Phang TL Neviani P Alvarez-Calderon F Eide CA O'Hare T Zaberezhnyy V Williams RT Druker BJ Perrotti D Degregori J Wnt/Ca2+/NFAT signaling maintains survival of Ph + leukemia cells upon inhibition of Bcr-Abl Cancer Cell 2010 18 74 87 20609354 10.1016/j.ccr.2010.04.025 PMC2904512

[B48] Coluccia AM Vacca A Duñach M Mologni L Redaelli S Bustos VH Benati D Pinna LA Gambacorti-Passerini C Bcr-Abl stabilizes beta-catenin in chronic myeloid leukemia through its tyrosine phosphorylation EMBO J 2007 26 1456 1466 17318191 10.1038/sj.emboj.7601485 PMC1817619

[B49] Ress A Moelling K Bcr is a negative regulator of the Wnt signalling pathway EMBO Reports 2005 11 1095 1100 16211085 10.1038/sj.embor.7400536 PMC1371031

[B50] Crews LA Jamieson CH Chronic Myeloid Leukemia Stem Cell Biology Curr Hematol Malig Rep 2012 7 125 132 22467334 10.1007/s11899-012-0121-6 PMC3342507

[B51] Chomel JC Turhan AG Chronic myeloid leukemia stem cells in the era of targeted therapies: resistance, persistence and long-term dormancy Oncotarget 2011 2 713 727 21946665 10.18632/oncotarget.333 PMC3248215

[B52] Leber B CML biology for the clinician in 2011: six impossible things to believe before breakfast on the way to cure Curr Oncol 2011 18 185 190 10.3747/co.v18i4.652 PMC3149551 21874109

[B53] Chomel JC Bonnet ML Sorel N Bertrand A Meunier MC Fichelson S Melkus M Bennaceur-Griscelli A Guilhot F Turhan AG Leukemic stem cell persistence in chronic myeloid leukemia patients with sustained undetectable molecular residual disease Blood 2011 118 3657 3660 21791426 10.1182/blood-2011-02-335497 PMC5162551

[B54] Helgason GV Young GA Holyoake TL Targeting chronic myeloid leukemia stem cells Curr Hematol Malig Rep 2010 5 81 87 20425400 10.1007/s11899-010-0043-0

